# Culture of Healthy Eating and Food Environments, Policies, and Practices in Regional New Zealand Schools

**DOI:** 10.3390/ijerph19116729

**Published:** 2022-05-31

**Authors:** Brittany Chote, Pippa McKelvie-Sebileau, Boyd Swinburn, David Tipene-Leach, Erica D’Souza

**Affiliations:** 1 Research and Innovation Centre, Eastern Institute of Technology, Napier 4112, New Zealand; pippa.mckelvie-sebileau@auckland.ac.nz (P.M.-S.); dtipene-leach@eit.ac.nz (D.T.-L.); 2 School of Population Health, University of Auckland, Auckland 1010, New Zealand; boyd.swinburn@auckland.ac.nz; 3 School of Future Environments, AUT University, Auckland 1010, New Zealand; e.dsouza@aut.ac.nz

**Keywords:** food environments, obesity prevention, nutrition policy, school meal programmes, food security

## Abstract

The school food environment plays an important role in shaping students’ dietary choices, which often influence future dietary behaviours. We surveyed primary and secondary schools in Hawke’s Bay, New Zealand, to measure the comprehensiveness and strength of food policies, describe the culture of food provision, and identify barriers to improving school food environments. Fifty-one schools were included in the final analysis, with 58.8% having a food policy, most of which used a generic template. Schools with food policies and those participating in the free and healthy lunch programme were more likely to have a strong culture around healthy eating. Common barriers to healthy eating were food outlets near school and resistance from students. Secondary schools reported facing more barriers to implementing healthy eating cultures, were more likely to use food as classroom rewards and to sell food to students, most of which was unhealthy. Hawke’s Bay schools participating in food provision programmes are successfully improving their food environments through improved culture and delivery of healthy food; however, more action is needed to strengthen the wording and guidance in food policies and reduce the provision of unhealthy food in schools before effective change can be achieved.

## 1. Introduction

A significant body of evidence now exists demonstrating the influence of the environment on dietary behaviours and associated outcomes [1,2,3]. Increasingly, food environments are considered unhealthy, with energy-dense, nutrient-poor processed foods being easily available, cheap, and widely advertised, leading to unhealthy diets, energy over-consumption, and growing obesity rates [4,5,6].

Children and adolescents in Aotearoa, New Zealand (NZ), have poor diets, with only 46% of 2–14-year-olds meeting the daily recommended intake of vegetables (at least two servings for 2–4-year-olds and at least three servings for 5–14-year-olds) and less than three-quarters meeting the fruit recommendation of at least two serves per day [7]. Young people in Hawke’s Bay—a region with a relatively large child and adolescent population, a significant proportion of Māori, and many living in areas of high deprivation—have even lower vegetable intakes, with only 13% of 9- and 13-year-olds meeting the daily vegetable guideline for their age [8]. Previous Hawke’s Bay research has also confirmed that Māori and those living in the least advantaged areas are at greater risk of carrying excess weight, the prevalence of overweight and obesity being 27% and 37%, respectively, in low-advantage schools, compared with 18% and 9%, respectively, in high-advantage schools [8,9].

The macro-environment (including the food industry, health and education systems, and society’s beliefs and attitudes) influence the micro-environments that people interact with on a daily basis, such as schools, workplaces, and homes [10]. School food environments are particularly significant given that students consume over one-third of their daily energy intake in this setting during the school week [11]. School meal programmes [12], school canteens, vending machines, and fundraising events [13,14] are important contributors to the food environment.

In NZ, there has been little monitoring of the school food environment and a lack of strong government policy, with National Administration Guideline 5b being the only national guideline covering healthy food environments for students [15]; this is due to the argument that dietary habits are a personal responsibility [16]. However, in response to the COVID-19 pandemic, a government-funded school lunch programme was rolled out nationwide in 2020 as part of the economic recovery package [17]. The programme, called Ka Ora, Ka Ako, provides healthy and free school lunches cooked at school or supplied by an external provider to all schools in the lowest 25% on the equity index [18] (lowest advantage) in a ‘whole of school’ approach. [17]. This is the largest intervention in children’s nutrition in NZ, and a full evaluation of the programme is required to determine whether goals are being met and to embed the programme within future education budgets [17]. In Hawke’s Bay, 40% of all students attend schools where Ka Ora, Ka Ako is operating.

The Nourishing Hawke’s Bay: He wairua tō te kai (there is life in this food) initiative is a community-based regional programme with the aim of improving food environments for children, especially Māori and other disadvantaged children. As part of this wider initiative and in response to the large presence of Ka Ora, Ka Ako in Hawke’s Bay, food environments in primary and secondary schools throughout the region were surveyed to meet the following objectives: to measure the comprehensiveness and strength of school food policies (i.e., whether they address all nutrition issues in a uniform and robust fashion); to describe the culture of food provision; to identify barriers to improving school food environments; to identify any patterns associated with participation in Ka Ora, Ka Ako; and to measure the impact of school advantage on food environments.

## 2. Materials and Methods

### 2.1. Study Design and Recruitment

The guiding thread of Nourishing Hawke’s Bay is a combination of Systems Thinking and mātauranga Māori (Indigenous knowledge). Systems Thinking has been increasingly applied to address complex public health issues in NZ and is proposed to be a good fit with mātauranga Māori, particularly in communities with large numbers of Indigenous people [19,20]. The focus of this paper is the cross-sectional online survey designed to measure different aspects of the school food environment. One representative (the principal, senior management, or health/nutrition teacher) took part from each eligible school within Hawke’s Bay between November 2020 and August 2021. The sampling frame included all schools listed in NZ’s Ministry of Education (MoE) school directory located in Hawke’s Bay (*n* = 127). Hospital schools, teen parent units, and correspondence schools were excluded because of their unique food environments. All remaining primary (including intermediate and full primary) and secondary schools (including composite schools that comprised both primary and secondary) in the Hawke’s Bay region were invited to participate either via email or during an in-person school visit by the researchers.

The survey was completed via Alchemer, an online survey software. Schools were also invited to provide their written food and beverage policy, if applicable, and researchers also searched school websites for available policies.

### 2.2. School-FERST Survey of Food Environments in Primary and Secondary Schools

We used the School Food Environment Review and Support Tool (School-FERST; see Supplementary File S1), a 26-item questionnaire, to survey primary and secondary school staff about their school food environments through a series of multi-choice, sliding-scale, and short answer questions. The tool consists of three sections. Part A covers nutrition policies and programmes (existence of nutrition and/or drinks policy, public availability, and whether students are allowed to leave school grounds during the school day and participation in food (provision of food) and nutrition (educational) programmes). Part B covers the provision and sale of foods and beverages, including foods for fundraising, celebrations, and rewards; the number of water fountains on site; methods of food sales on school grounds (tuck shops, canteens, lunch order system, fundraising, and vending machines); and the types of food sold to students. In this section, respondents were asked to indicate if any ‘red’ (unhealthy or ‘occasional’) [21] items were sold. The Ministry of Health (MoH) defines ‘red’ items as foods with poor nutritional value, high saturated fat and salt levels, and/or added sugars, which contribute to excess energy consumption and are often highly processed [21], for example, hot chips, cakes, doughnuts, pizza, hot dogs, confectionary, ice-cream, ice blocks, and sugar-sweetened beverages. The healthiness of food and/or beverages sold to students was estimated with a sliding scale to indicate the proportion of food and/or beverages for sale that were ‘red’ and the proportion that were ‘green’ (healthy or ‘everyday’) [21]. The MoH defines ‘green’ items as foods that are a good source of nutrition; are the basis of a healthy diet; are generally lower in saturated fat, salt, and added sugar; and are mostly whole or less processed [21], for example, sandwiches, wraps, salads, pasta with minimal cheese, tomato or vegetable-based sauce, fresh soups, rice or noodles with lean meat and vegetables, vegetable curry with rice, sushi, fresh fruit, low-fat milk, reduced-fat yoghurt, and wholemeal crackers with hummus/low-fat cheese. Part C covers school kaupapa (culture) around healthy eating. Respondents were asked to rate the culture of healthy eating at their school from very strong (policies in place; strong healthy food practices; students and parents strongly support the kaupapa of healthy food in school; nutrition is integrated across the curriculum) to very weak (no policy, considerable amounts of unhealthy foods provided/sold; healthy eating is a low priority for staff, students, and parents; limited nutrition education in the curriculum). They were also asked whether there were any barriers to implementing a healthy food and nutrition environment in their schools and, if there were, to identify the different types of barriers. Finally, open ended questions asked respondents to share the ways their schools were taking steps to create a healthy food and nutrition environment for students, staff, and whānau (families).

### 2.3. Nutrition Policy Analysis—Policy Food and Nutrition in Schools (Policy-FANS) Tool

When a school supplied their food policy or it was in the public domain (e.g., on school websites), the policies were rated using a modified version of the Wellness School Assessment Tool (Well-SAT, 2014) [22]. WellSAT was developed by the Rudd Centre for Food Policy and Obesity to evaluate the comprehensiveness and strength of school food and nutrition policies. As this 96-item tool was developed specifically for US schools, it was modified to suit the NZ context (WellSAT-NZ), the first iteration being a 40-item tool that was piloted and then used in a large-scale study in 2016 [23]. On the basis of the results from these initial surveys and published literature, the tool was shortened and modified further into the 10-item tool Policy-Food and Nutrition in Schools (Policy-FANS; see Supplementary File S2) that was used in this study. The four sections of the tool include Nutrition Education, Nutrition Standards for Foods and Beverages, Promoting a Healthy Food and Nutrition Environment, and Communication and Evaluation. Each item was rated as “0” (not mentioned), “1” (weak statement regarding the item), “2” (strong statement regarding the item), or N/A (for example, items NS1 “Addresses the implementation of the Nutrition Standards for foods and/or beverages sold to students during the school day” and NP2 “Addresses the promotion of healthy food and/or beverage items offered for sale through the school food service” if the school did not sell food or beverages). Two summary scores were given for each policy: policy comprehensiveness (maximum score of 10) was calculated by counting the number of items rated as “1” or “2” (0 = least comprehensive, 10 = most comprehensive), and policy strength (maximum score of 10) was calculated by counting the number of items rated as “2” (0 = weakest, 10 = strongest). For schools that did not sell food (i.e., NS1 and NP2 are N/A), the scores were calculated by dividing by eight and multiplying by 10.

Each written policy was analysed for comprehensiveness and strength by two reviewers independently. Inter-rater agreement was measured by the percentage agreement of the scores for each indicator across different policies (e.g., if schools were using the same generic template, only one version was considered). All discrepant scores were discussed with a third author to reach a final score.

### 2.4. School Demographics and Data Analysis

The type and socioeconomic classification for each school were obtained via the MoE’s school directory [24]. In NZ, the socioeconomic position of a school’s student community is rated by decile. Decile 1 schools are the 10% of schools with the highest proportion of students from low socioeconomic communities, while decile 10 schools are those with the lowest proportion of these students [25]. Survey data are reported as descriptives, with mean, 95% confidence intervals (95% CI), and ranges for primary and secondary schools separately. *t*-tests and ANOVA tests were used to identify between-group differences for quantitative scale variables. Subgroup differences were tested by Chi2 tests. A value of *p* < 0.05 was considered significant. The analysis was carried out in SPSS 27.

This study was approved by the Research Ethics and Approvals Committee of the Eastern Institute of Technology, ref 20/03.

## 3. Results

### 3.1. School Characteristics

Fifty-two responses to the School-FERST survey were received (43.7% response rate). One response was excluded, as school type (primary or secondary) was not provided, and no other identifying information was available. Of the 51 included schools, 38 (74.5%) were primary/intermediate schools (years 1–8), and 13 (25.5%) were secondary (years 9–12) or composite (years 1–13). Twenty-one schools were low-advantage (deciles 1–3, 41.2%); 18 were mid-advantage (deciles 4–7, 35.3%) and 12 were high-advantage (deciles 8–10, 23.5%). Approximately half (50.2%) were NZ European/Pākēha, 36.4% were of Māori ethnicity, 6.7% were Asian, and 5.5% were Pacific. These proportions are representative of the ethnicity of students enrolled in schools across the region [26].

Thirty-five (68.6%) schools participated in food programmes (food provision programmes designed to promote food security) and 37 (72.5%) participated in nutrition education programmes. All low-advantage and 61% of mid-advantage schools participated in at least one food programme (*p* < 0.001). The most common food programme was Ka Ora, Ka Ako [17] (45.1%), followed by KickStart Breakfast Club [27] (41.2%) and KidsCan [28] (39.2%). The most common nutrition programmes were Life Education Trust [29] (54.9%), Health Promoting Schools [30] (23.5%), and EnviroSchools [31] (19.6%). Primary schools were more likely to participate in nutrition education programmes (81.6%) compared with secondary schools (46.2%, *p* = 0.013), but there were no differences based on school advantage.

### 3.2. Policy Analysis

Only 30 (58.8%) schools indicated that they had a policy relating to food and nutrition, nine (17.6%) indicated their school did not, and 12 (23.5%) did not know. Of the schools that had a policy, less than a quarter (23.1%) reported that this policy was available for public access on the school website. No significant differences were found between school type or school advantage in reporting on the presence of a food policy.

Twenty-six school food and nutrition policies were received for analysis (seven low-, 12 mid-, and seven high-advantage schools). Twenty-three used a generic template from the website SchoolDocs [32]. Two versions of this template were recorded: (1) with a provision for water/unflavoured milk only in schools (*n* = 10) and (2) without specifying water/milk only (*n* = 13). For inter-rater reliability, only one version of each template was considered.

The inter-rater agreement for six indicators (NE2, NS2, NS3, NP1, CE1, and CE2) was over 85%, and for three indicators (NE1, NS1, and NP2), it was around 40%.

Policy comprehensiveness and strength scores are shown in Table 1; the mean comprehensiveness score was 6/10 (min–max: 3–7) and the mean strength score was 1.38/10 (min–max: 0–3). Mid-advantage schools scored lowest in policy comprehensiveness and strength (*p* < 0.05). There were no differences in policy comprehensiveness or strength between primary and secondary schools. Comprehensiveness and strength scores were higher (6.7 and 2) in templated policies with a provision for water only (*n* = 10) compared with templated policies without a provision for water only (5.8 and 1, respectively, *n* = 13) and individual school policies (4.3 and 1, respectively, *n* = 3).

### 3.3. Provision and Sale of Food and Beverages at School

Twelve schools (23.5%) indicated that they sold food and/or beverages to students on school grounds. Secondary schools were more likely to sell food to students, with eight (61.5%) secondary schools selling food through a canteen compared with four primary schools (10.5%, *p* < 0.001). Two-thirds of the canteens were run by the school. Mid-advantage schools were more likely to sell food and/or beverages to students on school grounds (50.0%) compared with low- (14.3%) and high- (0.0%) advantage schools (*p* < 0.01). With regard to the items for sale through the canteens, responses indicated that only two schools met the MoH recommendation of having at least 75% ‘green’ (healthy or ‘everyday’) foods for sale. No school reported having a vending machine on site.

The use of food and/or beverages for classroom rewards, celebrations, and fundraising is shown in Table 2. Forty schools (78.4%) used food and/or beverages for classroom celebrations (e.g., birthdays or end of term), 39 (76.5%) for fundraising, and 20 (39.2%) for classroom rewards. More secondary schools used food and/or beverages as classroom rewards (*p* < 0.01); however, there were no differences for school type and the use of food and/or beverages in classroom celebrations or for fundraising activities. Mid-advantage schools tended towards being more likely to use food in rewards, classroom celebrations, and fundraising, but because of the small numbers, these results were not statistically significant. For schools using food and beverages in fundraising, overall, the estimated ratio was 1:1 healthy to unhealthy; however, ratios ranged from 9:1 to 1:9 (healthy:unhealthy).

Four primary schools (10.5%) and four secondary schools (30.8%) said they allowed students to leave school grounds during the day to purchase food. While it appeared that mid-advantage schools were more likely to allow students to leave school grounds (27.8%) than low- (4.8%) or high- (16.7%) advantage schools, this was not statistically significant. Of note, two of these schools indicated that food outlets around the school were a barrier to providing a healthy food environment.

Over three-quarters of primary schools met the MoE’s recommendation of having at least one water fountain available at school for every 60 students; however, only one-quarter of secondary schools met the guideline (Table 3). Six schools (two primary and four secondary) had more than 100 students for each water fountain on school grounds, with one school having only one fountain for a roll of 305 students.

### 3.4. Perceived School Culture around Healthy Eating

Table 4 shows that when respondents were asked to rate the culture around healthy eating at their school (from very strong to very weak), 22 (44%) felt that the culture was very strong or strong. The mean culture score was higher (*p* < 0.05) for low-advantage (3.86) than mid- (3.24) and high-advantage schools (3.50). No differences were observed between primary and secondary. Schools participating in the government lunch programme (low-advantage) were more likely to have a very strong culture around healthy eating (26.1% for Ka Ora, Ka Ako schools vs. 3.7% for non-Ka Ora, Ka Ako schools), as were schools that had a nutrition policy (53.3% strong culture), compared with schools that did not have a policy (22.2% strong culture).

Fourteen schools (27.5%) reported barriers to implementing a healthy food environment in their school, 32 (62.7%) indicated no barriers, and five (9.8%) did not know. Common barriers reported are described in Table 5. It was more common for secondary schools to report barriers (46.2%) than primary schools (21.1%). Of note, reporting that unhealthy food outlets around the school were a barrier was most common for mid- (*n* = 6) and low-advantage schools (*n* = 3). No high-advantage schools experienced food outlets around the school as a barrier to implementing a healthy food environment in the school. High-advantage schools were more likely to identify resistance from parents as a barrier.

When asked to comment on the way schools were creating healthy food and nutrition environments, the most common theme was the provision of free and healthy food in schools, either through the Ka Ora, Ka Ako programme for low-advantage schools, free fruit baskets, KidsCan, Milk in Schools, or Parent Teacher Association cooked lunches once a week (12/35 comments). Two other strong themes were building healthy eating and, more generally, hauora (wellbeing) into the school focus, guidelines, policy, and curriculum:

“*Hauora is the focus of our Kura (school). Our aim is to have healthy kids who are connected to their culture*”

—principal, low-advantage primary school

Banning soft drinks from school and promoting water consumption through teachers modelling drinking from water bottles or being a ‘water-only’ school was mentioned by six schools:

“*We are a water only school. We encourage students to bring water bottles with them and model this*.”

—teacher, high-advantage primary school

Finally, respondents also noted that schools were promoting healthy eating through māra kai (vegetable gardens), banning confectionary, teachers modelling healthy eating, and using newsletters to communicate with parents.

## 4. Discussion

This regional study highlighted that Hawke’s Bay schools have the potential to create healthier food environments, and many have identified opportunities to do so, but overall policies and practices are inadequate. All low-advantage schools participated in food programmes, while primary schools were more likely to participate in nutrition education programmes compared with secondary schools. Where there were food policies in place, they were comprehensive but lacked strength, and there was a high reliance on generic policy templates. Low-advantage and Ka Ora, Ka Ako schools reported having a stronger food culture than other schools. However, low- and mid-advantage schools faced the barrier of unhealthy food outlets near school grounds. Secondary schools were more likely to have a canteen where the food sold was mostly unhealthy. The use of foods for classroom rewards, celebrations, and fundraising was widespread, with foods for fundraising continuing to be unhealthy. Primary schools tended to provide sufficient water fountains for their students, whereas only a quarter of secondary schools met the government requirements.

Schools in Hawke’s Bay were more likely to report having a food and nutrition policy in comparison with national data from the School-FERST survey administered in 2016 [23]. Unlike 2016, more high-advantage Hawke’s Bay schools reported having a policy compared with low-advantage schools. This might be due to the utilisation of the school policy subscription service SchoolDocs [32], which was very popular in this study, as it also was in 2016 [23]. Of the 26 policies submitted, 23 used the SchoolDocs template with little to no customisation. Each school embodies its own distinctive mix of students, staff, activity, and culture that needs to be considered during policy development and implementation. Such generic policies will have little effectiveness in improving the healthiness of food environments given the uniqueness between schools. School food policies in Hawke’s Bay also had higher comprehensiveness scores than those in the national study [23] but continued to lack strength because of their brief and suggestive nature, that is, they did not use strong or specific wording or include clearly stated goals and guidance. School food and nutrition policies are a crucial component of the school food environment because of their ability to impact the use of food broadly within the school [11]. Unfortunately, given the lack of engagement by schools to strengthen and customise policies, these benefits are rarely observed.

Mid-advantage schools had the lowest policy comprehensiveness and strength scores and were the most likely to sell food on site and fundraise using unhealthy foods. It is possible that these mid-advantage schools are missing out in comparison with high-advantage schools, which have stronger parent and community support, and low-advantage schools, which have substantial government funding. Mid-advantage schools would likely benefit from stronger direction from the government. Currently, food and nutrition guidelines for NZ schools are optional [21]; therefore, without strong direction from staff members or parents, food environments are not prioritised and do not support students to make healthy dietary choices [11]. A good example of this is the ‘water or milk only’ recommendation by the MoE. Sixteen of twenty-six food policies reviewed in this study did not include this recommendation. In April 2022, the MoE released a consultation paper proposing to ban the provision of unhealthy drinks in schools, which would mean that all state-funded schools in NZ would have to adopt this policy [33]. Further such policies would help support the creation of healthy school food environments.

Similar to the 2016 results [23], secondary schools were more likely to report facing barriers than primary schools, with resistance from students and parents/whānau among the top three barriers commonly faced. Low- and mid-advantage schools in Hawke’s Bay were also likely to report their efforts being hampered by surrounding unhealthy food outlets, which is analogous to NZ and international research [34,35,36]. Despite this, a number of primary and secondary schools allow students to leave grounds during the school day. Research shows that leaving school during lunch results in an increased likelihood of visiting local shops that sell unhealthy foods [37]. Such behaviours can be addressed by the presence of a strong policy restricting leave from school grounds [38,39,40]. Additionally, only one-quarter of secondary schools met the MoE water fountain recommendations [41]. Such patterns of inadequate drinking water provision have been noted internationally [42,43], despite evidence highlighting the positive impact on water intake from increased access to water in schools [44,45].

Qualitative data indicate that participation in food provision programmes, such as Ka Ora, Ka Ako, can play an important role in creating a healthy food and nutrition environment, while quantitative data also shows that school lunch programmes improve diet quality compared with habitual intake [46]. Yet, there is suggestion that the acceptance of such programmes by students and parents is mixed, as it is highly dependent on factors such as variety, choice, taste, the familiarity of foods, and portion size [47]. There are also issues with parental perceptions not aligning with the actual nutrition quality of the food [48]. However, the interim report of Ka Ora, Ka Ako cites demonstrated benefits to improving hunger and student wellbeing [49]. With its main aims of feeding and wellbeing, participation in such programmes can put nutrition on the radar and instigate a more positive attitude and school culture towards healthy eating as well as improved diet quality. Further evaluation and re-adjustment of Ka Ora, Ka Ako is needed by the government to ensure its presence and success in the future.

The sale of unhealthy food through fundraising continues to be a common practice in NZ schools [23,50], with a high proportion of Hawke’s Bay schools selling ‘red’ (unhealthy or ‘occasional’) items. Other practices, such as the use of foods and beverages for classroom celebrations and rewards, are also widespread, with the latter more common in secondary schools. Although this study was limited in its investigation, literature from North America illustrates that students consume mainly low-nutrient, energy-dense items during celebrations [51], that there is a lack of alternatives to using unhealthy food in fundraising, and that ‘special treats’ undermine the move towards healthier food environments in schools [52]. The variability in such practices between teachers and schools has resulted in scant research investigating its impact on health. Evaluating such practices is important, as it can negate other efforts in improving the school food environment.

### Strengths and Limitations

This study provides an insight into regional school food environments, building on previous NZ research. Although the survey response was moderate (43.7%), it is higher than the average response rate of 30% for online surveys [53] and is in line with international trends [54]. Additionally, as is the case with all self-reported data, there might be an element of social desirability bias. The shorter version of the School-FERST survey provided an updated snapshot of the healthiness of Hawke’s Bay school food environments in a representative sample including good coverage across low- to high-advantage schools and representative ethnicity coverage. To decrease the burden on busy schools, the survey was unable to obtain in-depth information to examine and validate variables such as policy implementation and observation of foods and beverages provided and sold. Further research focusing on the impact of school lunches on nutrient intake compared with habitual diet would also be useful.

## 5. Conclusions

Using the School-FERST survey and Policy-FANS tool, this study assesses the healthiness of school food environments and policies across the Hawke’s Bay region, providing a baseline for future Nourishing Hawke’s Bay and Ka Ora, Ka Ako evaluations. School food policies, though comprehensive, lacked strong and specific wording and were not designed to meet the needs of specific schools. Schools participating in Ka Ora, Ka Ako healthy school lunches reported a stronger culture around healthy eating, and schools considered the provision of free and healthy food to be a way of improving the school food environment. Unhealthy food outlets and resistance from students and parents were the most commonly reported barriers to implementing a healthy food environment, though these barriers differed by school advantage. Children in Hawke’s Bay continue to have poor dietary habits and high rates of obesity; therefore, more work is needed within schools and the government to move towards the creation of robust food policies and supportive school food environments to ensure that students have the opportunity to make the best possible dietary choices. The NZ Government has the levers to create strong policy, and they have started to pull these with the introduction of the free and healthy Ka Ora, Ka Ako school lunches, but more is needed to imbed this programme and other robust food environment policy into school life.

## Figures and Tables

**Table 1:** School food policy comprehensiveness and strength scores.

	Mean [95% CI]	Low-Advantage ^1^	Mid-Advantage	High-Advantage
Policy comprehensiveness score	6/10 [5.62–6.38]	6.43	5.50 *	6.43
Policy strength score	1.38/10 [1.10–1.67]	1.71	1.00 *	1.71

^1^ Low-advantage schools: deciles 1–3; mid-advantage schools: deciles 4–7; high-advantage schools: deciles 8–10. * *p* < 0.05.

**Table 2:** Hawke’s Bay schools using food and/or beverages in class and school activities.

	Primary Schools N (%)	Secondary Schools N (%)	Low-Advantage ^1^ N (%)	Mid-Advantage N (%)	High-Advantage N (%)
Uses food and/or beverages as classroom rewards	10 (26.3) ^2^	10 (76.9) ^2^	7 (33.3)	9 (50.0)	4 (33.3)
Uses food and/or beverages for classroom celebrations (e.g., birthdays)	28 (73.7)	12 (92.3)	16 (76.2)	15 (83.3)	9 (75.0)
Uses food and/or beverages for fundraising	28 (73.7)	11 (84.6)	13 (61.9)	17 (94.4)	9 (75.0)
Of those using food in fundraising, food includes unhealthy ‘red’ items	26/28 (92.9)	7/11 (63.6)	8/13 (61.5)	17/17 (100)	8/9 (88.9)

^1^ Low-advantage schools: deciles 1–3; mid-advantage schools: deciles 4–7; high-advantage schools: deciles 8–10; ^2^ Chi2. *p* < 0.01.

**Table 3:** Number of water fountains in Hawke’s Bay schools.

	Primary Schools N = 37 ^1^	Secondary Schools N = 12
Mean number of fountains (min–max)	7.8 (1–19)	7.2 (2–18)
Mean number of students per water fountain based on roll size (min–max)	48.8 (9–305)	92.0 (15–170)
Met the Ministry of Education guidelines (at least one fountain per 60 students)	29 (78.4 %)	3 (25.0 %)

^1^ One primary school and one secondary school were missing data for this question.

**Table 4:** Perceived culture around healthy eating in Hawke’s Bay schools.

	Schools ^1^ N (%) N = 50 *	Low-Advantage ^2^ N (%)	Mid-Advantage N (%)	High-Advantage N (%)
Very strong—policies in place, strong healthy food practices, students and parents strongly support the kaupapa of healthy food in school, nutrition is integrated across the curriculum	7 (14.0)	6 (28.6)	1 (5.9)	0 (0.0) **
Strong	15 (30.0)	6 (28.6)	3 (17.6)	6 (50.0)
Medium—some policies and practices support healthy food, mixed support for the kaupapa of healthy food by wider school community, nutrition education for some year levels	27 (54.0)	9 (42.9)	12 (70.6)	6 (50.0)
Weak	1 (2.0)	0 (0.0)	1 (5.9)	0 (0.0)
Very weak—no policy, considerable unhealthy foods provided/sold, healthy eating low priority for staff, students and parents, limited nutrition education in the curriculum	0 (0.0)	0 (0.0)	0 (0.0)	0 (0.0)

^1^ Primary and secondary schools combined; ^2^ Low-advantage schools: deciles 1–3; mid-advantage schools: deciles 4–7; high-advantage schools: deciles 8–10. * 1 school missing, ** *p* < 0.05.

**Table 5:** Barriers to implementing a healthy food and nutrition environment in Hawke’s Bay schools.

	Schools ^1^ N (%)
Efforts undermined by unhealthy food outlets around the school (e.g., local dairy/corner stores)	9 (17.6)
Resistance from students	9 (17.6)
Resistance from parents/whānau (family)	7 (13.7)
Loss of profits from the sale of less healthy foods and beverages	4 (7.8)
Lack of choice in the options provided by school food service (canteen)	4 (7.8)
Lack of convenience and difficulty in preparing fresh foods on-site	3 (5.9)
Other (e.g., cost of healthy food)	2 (3.9)
Resistance from Parents Teacher Association and/or Boards	0 (0.0)
Resistance from staff	0 (0.0)

^1^ Primary and secondary schools combined.

## Data Availability

Not applicable.

## Supplementary Materials

The following supporting information can be downloaded at: https://www.mdpi.com/article/10.3390/ijerph19116729/s1, Supplementary File S1: School Food Environment Review and Support Tool (School-FERST) Survey. Supplementary File S2: Policy Food and Nutrition in Schools (Policy-FANS) Tool.
