# Supplementary material for: Culture of Healthy Eating and Food Environments, Policies, and Practices in Regional New Zealand Schools
Source: Int J Environ Res Public Health. 2022 May 31;19(11):6729. doi: 10.3390/ijerph19116729 (PMC9180331; doi:10.3390/ijerph19116729)
Supplement: Supplementary file 1 [file ijerph-19-06729-s001.zip › ijerph-1731015-supplementary/File S1. School FERST survey.pdf]

School-FERST

Welcome

Page exit logic: Skip / Disqualify Logic
IF: #1 Question "**Consent Form**

Please confirm that you:

- have read the Participant Information Sheet (click here) and understand why your school has been invited.
- have had the opportunity to think about participation, ask questions and have them answered.
- agree to your school taking part to complete a self-review online questionnaire on school food environments (approximately 7-10 minutes).
- understand that participation is voluntary.
- understand that you are free to withdraw participation at any time without giving a reason.
- understand that the researchers guarantee confidentiality of your identity, and that school identification will be retained for feedback and support purposes as outlined in the Monitoring and Feedback Systems Information
- understand you will receive a letter with the results of the project at the end of project.
- understand that this consent form will be kept for 6 years, after which it will be destroyed

Do you agree to participate in the School-FERST Survey?" is one of the following answers ("No") **THEN:** Disqualify and display:
Thank you for taking the time to respond to our invitation to participate.

Thank you for taking part in the **Nourishing Hawke's Bay: He wairua tō te kai** project, and telling us a little bit about the food and nutrition at your school.

School principals are invited to complete this short survey, but you can delegate to another teaching or administrative staff or BoT member who has the appropriate knowledge about the food environment in your school.

Please have the following documents ready to upload (if available):

- School Food and Nutrition Policy
- School Food Service Menus (including Healthy Free Lunches programme, boarding house, lunch order-in system and/or canteen if applicable)

1. **Consent Form**

Please confirm that you:

- have read the Participant Information Sheet (click here) and understand why your school has been invited.
- have had the opportunity to think about participation, ask questions and have them answered.
- agree to your school taking part to complete a self-review online questionnaire on school food environments (approximately 7-10 minutes).
- understand that participation is voluntary.
- understand that you are free to withdraw participation at any time without giving a reason.
- understand that the researchers guarantee confidentiality of your identity, and that school identification will be retained for feedback and support purposes as outlined in the Monitoring and Feedback Systems Information
- understand you will receive a letter with the results of the project at the end of project.
- understand that this consent form will be kept for 6 years, after which it will be destroyed

Do you agree to participate in the School-FERST Survey?

○ Yes

○ No

Policies and Programmes

LOGIC Show/hide trigger exists.

2. **Does your school have a written policy that relates to food and nutrition?** (e.g. A formal policy set by the Board of Trustees to guide the school food environment)

○ Yes

○ No

○ Don't know

LOGIC Hidden unless: #2 Question "**Does your school have a written policy that relates to food and nutrition?**
(e.g. A formal policy set by the Board of Trustees to guide the school food environment)
" is one of the following answers ("Yes")

3. **Is the food and nutrition policy available for public access on the school website?**

- Yes
- No
- Don't know

4. **Does your school allow any students to leave school grounds during the school day?**
(e.g. senior students allowed to leave school during lunch to purchase food from local shops)

- Yes
- No
- Don't know

5. **Does your school currently participate in any of the following food programmes?**
(*tick all that apply*)

- ☐ The Free and Healthy School Lunches Programme
- ☐ Fonterra Milk in Schools
- ☐ Kick Start Breakfast
- ☐ Fruit in Schools
- ☐ KidsCan Food for Schools
- ☐ Other, please specify:
- ☐ Other, please specify:

6. **Does your school currently participate in any of the following nutrition programmes?**
(tick all that apply)

- ☐ Health Promoting Schools
- ☐ Life Education Trust
- ☐ Enviroschools
- ☐ 5+ A Day (Fresh Fruit and Vegetable Charitable Trust)
- ☐ Project Energize
- ☐ Heart Foundation Programmes (incl. Food for Thought)
- ☐ Garden to Table
- ☐ Other, please specify:
- ☐ Other, please specify:

Food and Beverages Provided and Sold

7. **How many water fountains/coolers are located on school grounds (including classrooms) and easily accessed by students?**

8. **Does your school use food and/or beverages as <u>classroom rewards</u>?**

- Yes
- No
- Don't Know

9. **Does your school use food and/or beverages for <u>classroom celebrations</u>?** (E.g. Birthdays or end of term)

- Yes
- No
- Don't know

LOGIC Show/hide trigger exists.

10. **Does your school use food and/or beverages for <u>fundraising activities</u>?**

- Yes
- No
- Don't know

LOGIC Hidden unless: #10 Question "**Does your school use food and/or beverages for fundraising activities?**
" is one of the following answers ("Yes")

11. Do you use any of the following Red / Occasional / Unhealthy items for fundraising?

hot chips, cakes, donuts, pizza, hot dogs, chocolate, lollies and other confectionary, *Moosies, Juicies,* ice-cream and ice blocks, fizzy drinks and sugar-sweetened juices, fast-food

- Yes
- No

LOGIC Show/hide trigger exists.

12. Are foods and/or beverages sold to students, on school grounds, during the school day?

(e.g. tuckshop/canteen run by school or operated by private provider)

- Yes
- No

LOGIC Hidden unless: #12 Question "**Are foods and/or beverages sold to students, on school grounds, during the school day?**
(e.g. tuckshop/canteen run by school or operated by private provider)
" is one of the following answers ("Yes")

13. **Which of the following sources sell foods and/or beverages to students during the school day?**
(*tick all that apply*)

- [ ] Canteen / Cafeteria / Tuckshop run by the school
- [ ] Canteen / Cafeteria / Tuckshop **not** run by the school (outsourced / private contractor)
- [ ] Lunch order system, please specify provider:
- [ ] Fundraising (e.g. the PTA sell lunch on Fridays)
- [ ] Vending machines
- [ ] Other source, please specify:

LOGIC Hidden unless: #12 Question "**Are foods and/or beverages sold to students, on school grounds, during the school day?**
(e.g. tuckshop/canteen run by school or operated by private provider)
" is one of the following answers ("Yes")

14. **Please use the sliding scale below to indicate the proportion of foods and/or beverages offered for sale that are clearly 'GREEN' (healthy, 'everyday'):**

This includes foods such as:

- sandwiches and wraps
- salads
- pastas with minimal cheese, tomato or vegetable based sauce
- fresh soups
- rice and noodles with lean meat and vegetables
- vegetable curry with rice
- sushi
- fresh-fruit
- low-fat milk, reduced fat yoghurt
- wholemeal crackers and hummus/low-fat cheese

0% ——— 100%
50%

LOGIC Hidden unless: #12 Question "**Are foods and/or beverages sold to students, on school grounds, during the school day?**
(e.g. tuckshop/canteen run by school or operated by private provider)
" is one of the following answers ("Yes")

15. **Please use the sliding scale below to indicate the proportion of foods and/or beverages offered for sale that are clearly 'RED' (unhealthy, 'occasional'):**

This includes foods such as:

- deep fried foods, including hot chips, chicken nuggets and dim sims
- hot pastries like sausage rolls and pies
- cakes, slices and sweet breads like donuts
- pizza, hot dogs and wedges
- chippes, and other assorted packaged chips (eg. *Doritos*)
- chocolate, lollies and other confectionary
- chocolate milk, including *Moosies*
- fizzy drinks and sugar-sweetened juices, sports and energy drinks, iced tea
- ice cream and ice blocks/*Juicies*
- Fast-food

0% 100%
50%

School Culture and Kaupapa

16. **How would you describe the culture/kaupapa around healthy eating at your school?**

Very strong	Strong	Medium	Weak	Very weak
(policies in place, strong healthy food practices, students and parents strongly support the kaupapa of healthy food in school, nutrition is integrated across the curriculum)		*(some policies and practices support healthy food, mixed support for the kaupapa of healthy food by wider school community, nutrition education for some year levels)*		*(no policy, considerable unhealthy foods provided/sold, healthy eating low priority for staff, students and parents; limited nutrition education in the curriculum)*
○	○	○	○	○

17. **Please rate the following statements:**

	Rating				
	Strongly Agree	Agree	Neither agree nor disagree	Disagree	Strongly Disagree
**Nutrition and healthy eating are highly prioritized at our school** *(incorporated into school vision, policy and practices)*	○	○	○	○	○
**Nutrition education is substantial and integrated across the curriculum** *(all year levels and not a one-off delivery)*	○	○	○	○	○
**Staff consistently act as role models for healthy eating** *(teachers meals are healthy; workshops and meetings cater only healthy options)*	○	○	○	○	○
**The school frequently communicates with parents and whānau about nutrition and healthy eating** *(enrollment information, newsletters, website and the school app)*	○	○	○	○	○
**The Board of Trustees and PTA share a strong collective vision around hauora/health at the school**	○	○	○	○	○
**The principal and staff share a strong collective vision around hauora/health at the school**	○	○	○	○	○

LOGIC Show/hide trigger exists.

18. **Are there any <u>barriers</u> to implementing a healthy food and nutrition environment in your school?**

- ○ Yes
- ○ No
- ○ Don't know

LOGIC Hidden unless: #18 Question "**Are there any <u>barriers</u> to implementing a healthy food and nutrition environment in your school?**
" is one of the following answers ("Yes")

19. **Does your school experience any of the following barriers?**
(tick all that apply)

- ☐ Resistance from students
- ☐ Resistance from parents/whānau
- ☐ Resistance from staff
- ☐ Resistance from PTA and/or Board of Trustees
- ☐ Loss of profits from the sale of less healthy foods and beverages
- ☐ Efforts undermined by unhealthy food outlets around the school (e.g. the local dairy)
- ☐ Lack of convenience and difficulty in preparing fresh foods on-site
- ☐ Lack of choice in the options provided by school food service (canteen) provider
- ☐ Other, please specify: ____________
- ☐ Other, please specify: ____________

20. **Please describe any other ways in which your school is taking steps to create a healthy food and nutrition environment for students, staff and whānau:**

21. **Please describe anything else relevant but not covered in this survey about the food environment at your school:**

A little bit about you

22. **Please indicate who filled out the questionnaire:**

- Principal
- Senior Management (Deputy Principal, Head of Department)
- Teaching Staff
- Administrative / Support staff (e.g. Office Manager, Receptionist, Food Service Staff, School nurse, Librarian, etc.)
- Board member / Trustee
- Parent / Whānau
- Other, please specify:

23. **Name of school**

(To provide individual feedback on your school's food environment. Your school will not be identified in any outputs)

24. **School suburb**

(To provide comparative feedback with schools in your decile and region)

25. **Please enter your email address so that we may send the results directly to you**

26. **Number of students currently enrolled at your school:**

LOGIC Hidden unless: #2 Question "**Does your school have a written policy that relates to food and nutrition?**
(e.g. A formal policy set by the Board of Trustees to guide the school food environment)
" is one of the following answers ("Yes")

27. **If available, please attach a copy of your school's food and nutrition policy.**

Alternatively you can email a copy of your policy to: nourishinghb@eit.ac.nz

Browse...

LOGIC Hidden unless: #12 Question "**Are foods and/or beverages sold to students, on school grounds, during the school day?**
(e.g. tuckshop/canteen run by school or operated by private provider)
" is one of the following answers ("Yes")

28. **If available, please attach a copy of your school food service menu** (canteen/tuckshop, free school lunches or lunch order system)

Alternatively you can email a copy of your menu to: nourishinghb@eit.ac.nz

Browse...

Thank You!

Ngā mihi nui ki a koe!

Thank you for taking part in the School-FERST survey, part of the Nourishing Hawke's Bay: He wairua tō te kai project. Your response is very important to us. We will be in touch and email your results shortly.

If you have any questions, please email us: nourishinghb@eit.ac.nz
