# Supplementary material for: Culture of Healthy Eating and Food Environments, Policies, and Practices in Regional New Zealand Schools
Source: Int J Environ Res Public Health. 2022 May 31;19(11):6729. doi: 10.3390/ijerph19116729 (PMC9180331; doi:10.3390/ijerph19116729)
Supplement: Supplementary file 1 [file ijerph-19-06729-s001.zip › ijerph-1731015-supplementary/File S2. Policy FANS tool 2021.pdf]

Policy for Food And Nutrition in Schools (Policy-FANS)

The Policy-FANS tool is based on an adapted version of the WellSAT-NZ tool utilised in the School-FERST study (2016).

The initial version of the WellSAT-NZ tool is an adapted version (to ensure relevancy to New Zealand contexts) of the Wellness School Assessment Tool (WellSAT) developed in the United States by researchers funded by the Robert Wood Johnson Foundation. This validated assessment tool provides a standardized measure to evaluate school food and nutrition policies using quantitative methods.

How to Rate School Food and Nutrition Policy Statements

School Wellness policies are evaluated based on the degree to which they address 10 policy items, which are categorised into four sections. The sections include Nutrition Education, Nutrition Standards for Foods and Beverages Provided and Sold, Promoting a Healthy Food and Nutrition Environment, and Food and Nutrition Communication and Evaluation.

For each of the 10 policy items, policy statements are to be rated "0," "1," or "2," using the definitions below. Three items may be rated "NA" if the policy statement does not apply. This evaluation tool lists each policy item followed by an explanation of the item and examples of "1" and "2" statements.

		Explanation
0	= Not Mentioned	The item is not addressed in the text of the policy.
1	= Weak Statement	Assign a rating of "1" when the item is mentioned, <u>*but*</u><br>• The policy will be hard to enforce because the statement is *vague*, *unclear*, or *confusing.*<br>• Statements are listed as *goals*, *aspirations*, *suggestions, objectives,* or *recommendations.*<br>• There are *loopholes* in the policy that weaken enforcement of the item, such as a *disclaimer* that states that policies are non-enforceable and/or subject to change without notification.<br>• The policy mentions a *future plan to act* without specifying when the plan will be established.<br>Words often used include*: **may, can, could, should, might, encourage, suggest, urge, some, partial, make an effort,*** and ***try***.
2	= Meets / Exceeds Expectations	Assign a rating of "2" when the item is mentioned, and it is clear that the child care center is committed to making the item happen because:<br>• The item is described using specific language (e.g., a concept followed by concrete plans or strategies for implementation).<br>• Strong language is used to indicate that action or regulation is required, including: ***shall, will, must, have to, insist, require, all, total, comply*** and ***enforce.***<br>• A centre is unable to enforce an item, but the goal is clearly stated (e.g., "teachers eat the same foods served to children").<br>• Statements in parent handbooks that are preceded with "please" (e.g., "please do not bring in sweets or dessert foods for your child's lunch") are acceptable for a rating of a "2."<br>• Instructional statements in the staff manual (e.g., "do not use food as reward or punishment") are acceptable for a rating of a "2."

**Evaluating hint**	One method for deciding between a rating of a “1” and a "2” is to consider the scenario of a parent approaching a school principal or Head Teacher to discuss an issue. If the policy is ambiguous on how the school should handle the issue at hand, rate the item as a "1." If the written policy gives clear guidance about how to decide whether the school complies with the policy, rate the item as a "2."

How to Score School Food and Nutrition Policies

Policies are scored to determine Comprehensiveness and Strength. Comprehensiveness reflects the proportion of item topics that are simply mentioned. Strength reflects the proportion of items that are addressed with *specific* and *directive* language.

Score	Explanation
**Total Comprehensiveness Score**	Total comprehensiveness is calculated by counting the number of items in each section rated as “1” or “2,” and dividing this number by 10
**Total Strength Score**	Total strength is calculated by counting the number of items in each section rated as “1” or “2,” and dividing this number by 10

***There are two items (NS1, NP2) for which it is possible to receive a rating of “NA.” Items rated “NA” should not be included in the total number of items used for scoring a particular section. This will also change the denominator, that is, instead of 10 it will change to the number of indicators relevant, 8.**

Section A. Nutrition Education

#	Item	Rating Guidance	
NE1	**Addresses the inclusion of nutrition education in the teaching curriculum for all grades.**	**Note:** This item does not include informal nutrition education during mealtimes.	
		0	Not mentioned.
		1	**Either of the following:**<br>• Describes a general health and wellbeing curriculum or lesson plan, and implies but does not ensure, that lessons address nutrition specifically<br>• Suggestions that students receive nutrition education and/or healthy eating will be promoted in the curriculum<br>• Mention of an internal school health program that implies but does not ensure nutrition education is addressed specifically<br>**Examples:** • *"The health education curriculum is designed to promote healthy eating and activity"*<br>• *"Staff will try incorporate nutrition education into the already existing teaching curriculum where appropriate"*<br>• *"Students learn about different kinds of foods through nutrition education included in the curriculum"*
		2	**Requires that**<br>• Nutrition education is provided to the whole school.<br>**Examples:** • *"The school curricula will include lessons on nutrition for students from all year levels"*<br>• *"The health education curricula will include lessons on nutrition and healthy eating….."*

#	Item	Rating Guidance	
NE2	**Addresses the provision of nutrition education training for teachers.**	0	Not mentioned.
		1	**Either of the following:**<br>• Suggestions for the provision of nutrition education for staff<br>• Mentions nutrition training for staff but no specific activities/tasks identified<br>**Example:** *"It is recommended that teaching staff participate in health education training, which includes training on topics such as nutrition…"*
		2	**Requires**<br>• Provision of nutrition education training for staff, which can include training in facilitating or planning nutrition-related activities.<br>**Example:** *"All teaching staff must undertake annual training on nutrition education curriculum and activities for students…"*

Section B. Nutrition Standards for Food and Beverages Provided and Sold

#	Item	Rating Guidance	
NS1		0	Not mentioned.
		1	**Vague and/or suggested.**<br>**Examples:**

	**Addresses the implementation of the Nutrition Standards for foods and beverages <u>sold to students during the school day</u> (e.g. school canteen/cafeteria, lunch order-in system, vending machines)**		• *"All food and beverages provided and sold to students should comply with the Ministry of Health's Food and Nutrition Guidelines."*<br>• *"It is recommended that all foods and beverages sold at school comply with recommendations form Fuelled4Life"*
		2	**Requires**<br>• Compliance and specific criteria/standards mentioned.<br>**Examples:** • *"All foods and beverages sold to students must comply with the Ministry of Health Food and Nutrition Guidelines"*<br>• *"Only 'everyday' and 'sometimes' foods will be served at the school canteen"*<br>• *"Foods that are categorized as 'occasional' by Fuelled4Life including fizzy drinks, fruit drinks, flavoured milk, lollies, chocolate, sugary cereals, and/or hot chips are not allowed to be served or consumed at our school."*
NS2	**Addresses implementation of the Nutrition Standards for <u>classroom rewards, celebrations, school events, and/or special occasions, and fundraising activities.</u>**	0	**Either of the following:**<br>• Not mentioned.<br>• Policy allows traditional party foods (e.g., cake, confectionery, or biscuits) without stating any limits<br>• Does not mention nutrition standards for food sold for fundraising, or mentions the use of unhealthy food for fundraising.<br>**Example:** *"The fundraising activities may include sausage sizzles, confectionery sales, bake sales, and special events."*
		1	**Any of the following:**<br>• Vague, suggested, or weakened by exceptions such as staff discretion.<br>• Mentions compliance to one or more standards but does not specify how it will do so<br>• Suggests or recommends the use of non-food alternatives for celebrations and classroom rewards<br>**Example:** *"Healthier food options will be encouraged at school camps"*<br><br>**Any of the following:**<br>• Vague and/or suggested, or weakened by staff discretion<br>• Mentions compliance to one or more standards but does not specify how it will do so<br>• Regulations of foods/beverages sold for fundraising only apply to a limited group of items or only to food sales on school grounds (but excludes activities off the school premises).<br>**Example:** *"There will be no sausage sizzles or food sales for fundraising allowed during the school day."*
		2	**Requires**:<br>• Prohibits the use of foods/beverages as classroom rewards<br>• Compliance to nutrition standards <u>and</u> that specific criteria is mentioned<br>**Examples:** *"Staff are not allowed to use food and beverages as classroom rewards"*<br>*"All foods and beverages provided and sold at school events must comply with the Food and Beverages Classification System"*<br><br>**Either of the following:**<br>• Prohibits the sale of foods/beverages for fundraising.

			• Compliance to nutrition standards and that specific criteria is mentioned<br>**Example** *"Only 'everyday' and 'sometimes' items can be used for fundraising initiatives"*
NS3	**States that beverage provision and sale for students is milk and water only (no sugary drinks at any time).**	0	Not mentioned.
		1	Vague and/or suggested<br>**Example:** "*Water is the preferred choice"*
		2	Requires that only plain reduced or low-fat cow's milk, and soy/rice milk will be provided and/or sold to students.<br>**Example:** *"Only green, yellow or light-blue top, plain cow's milk is available at the canteen."*

Section 3. Promoting a Healthy Food and Nutrition Environment

#	Item		Rating Guidance
NP1	**Encourages teachers to be role models for healthy eating, including staff consumption of foods and/or beverages meeting the Nutrition Standards.**	0	Not mentioned.
		1	**Either of the following:**<br>• Vague and/or suggested.<br>• Mentions teachers promoting and encouraging healthy eating<br>**Example:** *"Teachers are encouraged to adhere by the school nutritional standards"*
		2	**Requires:**<br>• Staff to model healthy eating behaviours during all interactions with students<br>• Staff to comply with the schools (or standards such as NS1) prescribed nutritional standard<br>**Example**: *"Teachers must adhere to the same standards prescribed for students"*
NP2	**Addresses the promotion of healthy foods and beverages offered for sale through the school food service**	0	Not mentioned.
		1	**Vague or suggested:**<br>• Healthy foods will be promoted in the canteen (but does not mention how)
		2	Required that schools identify and describe actionable steps to promote the sale of healthy food and beverage items.<br>**Example:** *"Healthy foods will be placed at the front of the canteen"*<br>*"Healthy food and beverages will always be available at a cheaper price than other items ."*

Section D. Food and Nutrition Communication and Evaluation

Code	Criterion	Score	Description
CE1	**Addresses the communication of the school food and nutrition policy to students, staff and parents.**	0	**Either of the following:**<br>• Not mentioned.<br>• Only mentions that parents can visit the school at any time<br>**Example:** *"We have an open door policy. Parents are welcome at any time."*
		1	**Any of the following:**<br>• Vague and/or suggested.<br>• Mentions that changes in the policy should be notified to students, staff and parents but does not specify how this will occur<br>**Example:** *"Students and parents will be notified if there are any changes to school policies"*
		2	Required that schools notify all member part of the school community of any changes in school policies, and outlines the methods through which this will take place<br>**Example:** *"Students and parents will be notified via email and the school newsletter when policies are modified, reviewed and updated"*
CE2	**Addresses the provision of nutrition education for parents.**	0	Not mentioned.
		1	**Either of the following:**<br>• Vague and/or suggested.<br>• Describes the provision of nutrition education for parents but does not mention specific methods or activities.
		2	**Requires either of the following:**<br>• Provision of at least one nutrition education activity for parents, such as workshops or information via newsletters.<br>• Parents are encouraged and notified to access nutrition education materials on the school website/in the enrolment handbook<br>**Example:** "*The school will host a whanau night once a term to cover topics and discussions on healthy eating and nutrition for students and families".*<br>*"Nutrition education information will be provided on the school website/school newsletters"*
CE3	**Specifies a suitable timeframe for revising and updating the school food and nutrition policy.**	0	Not mentioned, or the stated date for revision has passed or is blank.
		1	**Either of the following:**<br>• Vague and/or suggested.<br>• Mentions revisions (e.g., changes, additions, or recommendations) to policies or program but does not refer specifically to the health/wellness policies or programs.<br>• Has a specific review date (e.g. review by February 2016) but does not specify the process for review
		2	Identifies a plan for revising the health, wellness, or nutrition policies or programs.<br>**Examples:** *"An annual review conducted by senior management and a registered dietitian will make recommendations to ensure that the policy is fulfilling school vision and goals"*

Policy for Food and Nutrition in Schools (Policy-FANS) Score Sheet

School name: **Date of Assessment:**

The *Policy-FANS Score Sheet* summarizes a school's food and nutrition policy scores based on four sections containing a total of 10 policy items. Each item is rated as "0" (not mentioned), "1" (weak statement regarding the item), "2" (strong statement regarding the item) or NA (items NS1 and NP2) if the school does not sell food or beverages. **Comprehensiveness** (score out of 10) is calculated by counting the number of items rated as "1" or "2". **Strength** (score out of 10) is calculated by counting the number of items rated as "2." For schools which do not sell food (ie NS1 and NP2 are NA) the scores are calculated by dividing by 8 and multiplying by 10.

'Nutrition Standards' refers to the Ministry of Health's Healthy Food and Drink Guidance (2020).

	Nutrition Education	Rating
NE1	**Addresses the inclusion of nutrition education in the teaching curriculum for all grades.**	
NE2	**Addresses the provision of nutrition education training for teachers.**	

	Nutrition Standards for Food and Beverages Provided and Sold	Rating
NS1	**Addresses the implementation of the Nutrition Standards for foods and beverages sold to students during the school day (e.g. school canteen/cafeteria, lunch order-in system, vending machines)**	
NS2	**Addresses implementation of the Nutrition Standards for classroom rewards, celebrations, school events, and/or special occasions, and fundraising activities.**	
NS3	**States that beverage provision and sale for students is milk and water only (no sugary drinks at any time).**	

	Promoting a Healthy Food and Nutrition Environment	Rating
NP1	**Encourages teachers to be role models for healthy eating, including staff consumption of foods and/or beverages meeting the Nutrition Standards.**	
NP2	**Addresses the promotion of healthy food and/or beverage items offered for sale through the school food service.**	

	Food and nutrition communication and evaluation	Rating
CE1	**Addresses the communication of the school food and nutrition policy to students, staff and parents.**	
CE2	**Addresses the provision of nutrition information for parents.**	
CE3	**Specifies a suitable timeframe for revising and updating the school food and nutrition policy.**	

Overall School Policy Scores

Total Comprehensiveness score /10

Total Strength score /10
